# What should we expect when two myositis-specific antibodies coexist in a patient

**DOI:** 10.1186/s40001-023-01363-5

**Published:** 2023-10-12

**Authors:** Yiming Zheng, Yawen Zhao, Hongjun Hao, Zhaoxia Wang, Feng Gao, Wei Zhang, Yun Yuan

**Affiliations:** Neurology Department, Peking University First Hospital, No. 8 Xishiku Street, Xicheng District, Beijing, 100034 China

**Keywords:** Myositis-specific antibodies, Idiopathic inflammatory myopathy, Anti-SRP antibody

## Abstract

**Background:**

The coexistence of two myositis-specific autoantibodies (MSA) is considered extremely rare. We describe three patients with both anti-signal recognition particle (SRP) antibodies and another MSA in serum.

**Methods:**

We performed a retrospective clinical data collection and follow-up studies of the clinical manifestations and treatment outcome of three patients positive with anti-SRP antibodies and other MSAs. IgG antibodies against MSAs were detected using commercial line immunoblot assay.

**Results:**

The tests of MSA showed positive result of anti-SRP antibodies and another one MSA including anti-TIF1-γ, anti-Jo1, or anti-EJ antibodies, respectively. The proximal muscle weakness appeared in 2 patients; interstitial lung disease presented in 2 patients. The serum CK level was elevated in 1 patient. The muscle biopsy showed necrotizing myopathy in 1 patient and deposition of membrane attack complex on scattered myofibers in the other one patient. One of the two patients with interstitial lung disease died because of respiratory failure. One patient had completely improved and the other one showed partial remission after immunosuppressive therapy.

**Conclusions:**

The patients with anti-SRP antibodies co-occurred with the other MSA may have various clinical characteristics. The clinicopathological phenotypes of these patients seem to be mainly caused by one of the MSAs, namely the responsible antibody.

## Introduction

Many autoantibodies were found in patients with idiopathic inflammatory myopathy (IIM), which have been divided into myositis-specific antibodies (MSAs) and myositis-associated antibodies (MAAs). MSAs can help identify distinct clinical phenotypes of IIM, which have been used for differential diagnosis [1]. Antibody against signal recognition particle (SRP) is a common autoantibody in immune-mediated necrotic myopathy (IMNM) [2]. IMNM with anti-SRP antibody is characterized by severe muscle weakness, very high levels of creatine kinase (CK), relatively infrequent extra-muscular involvement including rashes and interstitial lung disease, and refractory to steroid treatment [1–3]. Anti-transcriptional intermediary factor 1-γ (TIF1-γ) antibody is a common autoantibody in dermatomyositis, characterized by classic dermatomyositis rashes and mild muscle involvement. Anti-Jo1 is a common autoantibody in anti-synthetase syndrome, characterized by interstitial lung disease, muscle weakness and skin involvement. Anti-EJ is a rare autoantibody in anti-synthetase syndrome, which is characterized by interstitial lung disease and muscle weakness, but no skin involvement [1]. An intriguing aspect of MSAs is that the coexistence of two MSAs in the same individual is uncommon [4–10]. The characterizations of these patients with more than one MSAs have not been well described. Here, we report the clinical manifestations of three cases with multiple MSAs to discuss whether the clinicopathological phenotypes of these patients are superimposed or dominated by one responsible antibody.

## Materials and methods

### Clinical data (Table 1)

**Table 1:** The clinical manifestations of patients with anti-SRP antibodies and another MSA

	Age/gender	Muscle weakness	Skin lesion	Raynaud’s phenomenon	Arthritis	Mechanics hands	Tumor	ILD	CK	MSAs	Muscle biopsy	Therapy	Outcome
Patient 1	33/F	+	−	−	+	−	−	−	Normal	SRP, TIF1-γ	Mild	GC	Recover
Patient 2	78/F	−	−	−	+	−	−	+	Normal	SRP, Jo1	NA	GC + CTX	Partial remission
Patient 3	74/M	+	−	−	−	−	−	+	3750	SRP, EJ	NAM	GC + IVIG	Death
REF4 [5]	70/F	+	+	+	−	NA	−	+	1100	SRP, Jo1	PM	GC + CTX	Mild improvement
REF5 [6]	37/F	+	−	+	−	−	−	+	21,808	SRP, PL12	NAM	GC + IVIG + CTX	Partial remission
REF6 [7]	61/M	+	+	−	−	−	Gastric cancer	+	5685	SRP, Jo1	DM	Gastrectomy, GC	Death
REF7 [8]	33/F	NA	NA	NA	+	NA	NA	+	NA	SRP, PL12	NA	NA	NA
REF8 [9]	46/F	+	−	+	−	−	−	+	3500	SRP, PL12	Mild	GC	Greatly improved
REF9 [10]	19/M	+	−	−	−	−	−	−	311	SRP, Mi2	NAM	GC, AZA/MMF	Good response

F, female; M, male; NA, not available; ILD, interstitial lung diseases; CK, creatine kinase; MSAs, myositis-specific autoantibodies; NAM, necrotizing autoimmune myositis; PM, polymyositis; DM, dermatomyositis; GC, glucocorticoids; CTX, cyclophosphamide; AZA, azathioprine; MMF, mycophenolate mofetil; IVIG, intravenous immunoglobulin

#### Patient 1

A 38-year-old woman presented to our neurology department due to weakness in the proximal lower limbs for 1 month. She also had a history of unexplained intermittent joint swelling and pain, involving the wrist and knee joints for 5 years. No dysphagia, dyspnea, fever or rash was found. She has no history of taking statins. Physical examination revealed symmetrical proximal lower limb weakness (grade 4/5 (MRC)), without skin rashes. The erythrocyte sedimentation rate was slightly elevated (55 mm/h, normal range: 0–15 mm/h). The CK levels, rheumatoid factor, tumor markers and computed tomography (CT) scan of her lung were all normal. The electromyography revealed myopathic changes in her proximal muscles of the lower limbs. Serum antinuclear antibody (ANA) was detected as 1:100 (speckled nuclear and speckled cytoplasmic). The anti-Sm antibody was positive (+ + +). She underwent a muscle biopsy in left quadriceps and a serum myositis antibody test.

#### Patient 2

An 81-year-old woman was admitted because of a 3-month history of persistent nonproductive cough and progressive shortness of breath. She also had joint pain and intermittent fever. She denied rash and Raynaud phenomenon. No muscle weakness was noticed. The past history was noncontributory to the present symptoms. Physical examination showed Velcro rales on bilateral lower part of the lung. No mechanic’s hand was found. The ANA was positive (1:3200, nucleolar and cytoplasmic). The CK levels, rheumatoid factor, cyclic citrullinated peptide antibodies, anti-double-stranded DNA (dsDNA) and anti-extractable nuclear antigen (ENA) antibodies were all normal. The arterial blood gases test showed mild hypoxemia. The pulmonary function test showed a restrictive defect and a severe reduction in the diffusing capacity. The CT of the chest revealed the severe changes consistent with interstitial lung diseases. No cancer was found after screening tests. She underwent a serum myositis antibody test.

#### Patient 3

A 74-year-old male had shortness of breath with persistent nonproductive cough for 6 months. The symptoms became more serious with intermittent fever (the highest temperature reached up to 39 °C) 1 month ago before hospitalization. He had symmetric proximal limbs weakness and myalgias 1 week ago with difficulty to climb stairs and lift his arms. He also had dysphagia. He did not have rash or joint pain. He had a history of hepatitis B and tuberculosis before. On physical examination, there were Velcro rales on bilateral lung and severe symmetric proximal limb weakness (MRC 2–4/5) with weakness of the neck flexors (MRC 2/5). The CK levels were abnormal (1803–3750 IU/L, normal range: 25–195 IU/L). The ANA was positive (1:320, cytoplasmic), and the dsDNA and ENA antibodies were all negative. The arterial blood gases test revealed hypoxemia and carbon dioxide retention. The chest CT showed the changes of interstitial lung disease and a small amount of pleural effusion on both sides. He underwent a muscle biopsy in left deltoid and a serum myositis antibody test.

### Detection of MSAs and MAAs

IgG antibodies against 11 (including seven MSAs: Jo1, PL7, PL12, EJ, OJ, Mi2 and SRP; and four MAAs: Ku, PM-Scl100, PM-Scl75 and Ro52) or 16 (including twelve MSAs: Mi-2α, Mi-2β, TIF1-γ, MDA5, NXP2, SAE1, Jo1, PL7, PL12, EJ, OJ and SRP; and four MAAs: Ku, PM-Scl100, PM-Scl75 and Ro52) different cytoplasmic/nuclear antigens were detected using commercial line immunoblot assay (Euroline Myositis Profile 3 or 4, respectively) according to the manufacturer’s instructions. Band intensity was reported relative to grey scale intensity measured on a CanonScan LIDE 100 Scanner (Canon, Japan) using Line Scan scanning software (Euroimmun). The manufacturer’s recommended cut-offs of a band intensity of 10 was used to analysis the results, and the grading was as follows: 11–25, ( +), positive; 26–50, (+ +), positive; 51–256 (+ + +), strong positive [11, 12]. The ANA was detected using the indirect immunofluorescence assay on HEp-2 cells (HEp-2 IFA).

### Muscle biopsy

Muscle biopsies were performed in patient 1 and patient 3, for routine histological, enzyme histochemical, and immunohistochemical staining. First antibody against CD3, CD4, CD8, CD20, CD68, major histocompatibility complex 1 (MHC-I), and membrane attack complex (MAC) were stained.

The study was approved by the ethics committee of Peking University First Hospital and all patients gave informed consent.

## Results

**Fig. 1:**
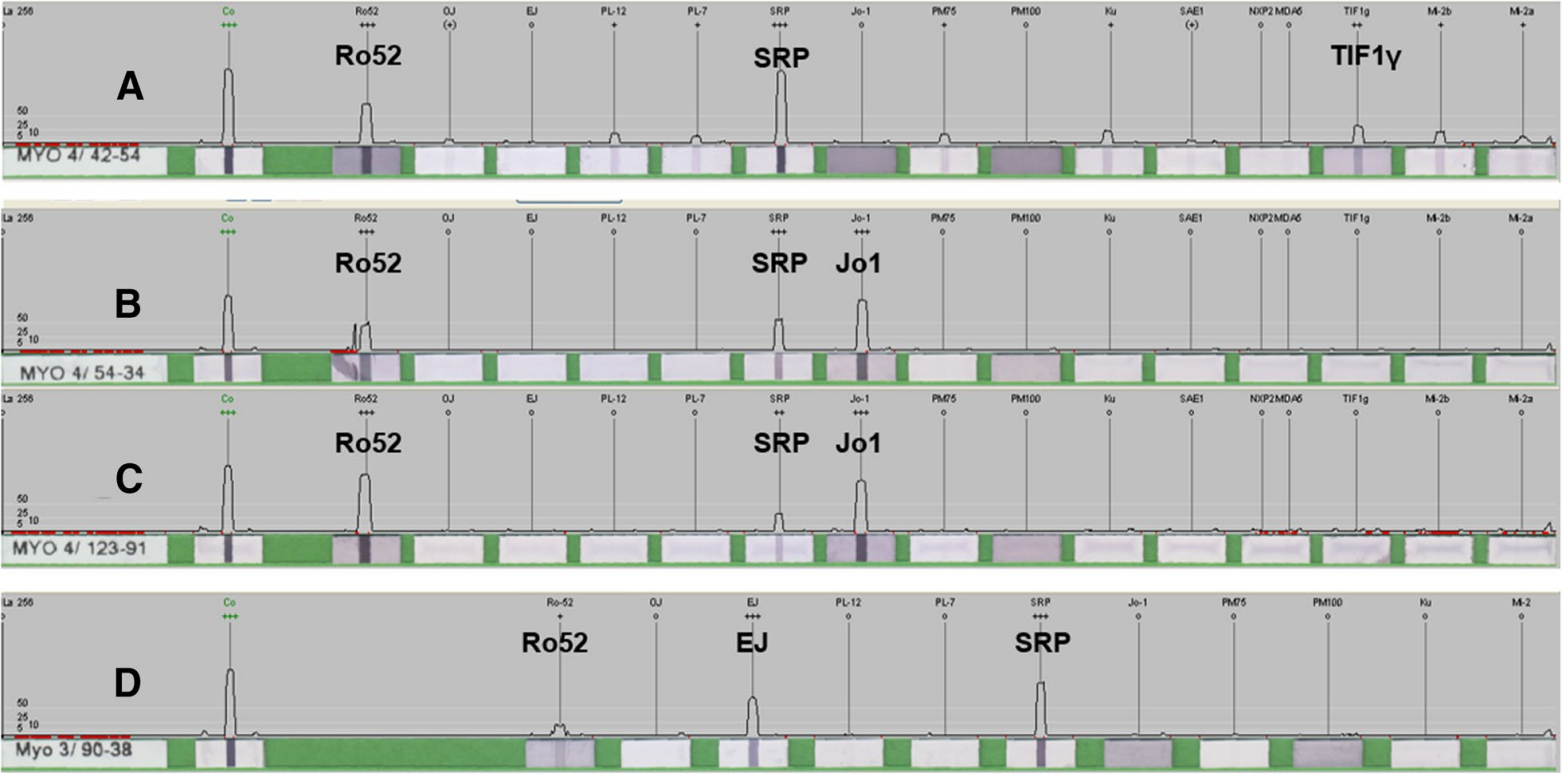
MSAs and MAAs detected in three patients. **A** showing positive results of anti-Ro52 (+ + +), SRP (+ + +) and TIF1-γ (+ +) in patient 1; **B**, **C** showing positive anti-Ro52 (+ + +), SRP (+ +  + / + +) and Jo1 (+ + +) in patient 2 in two tests; **D** showing positive anti-Ro52 ( +), SRP (+ + +) and EJ (+ + +) in patient 3

All patients had anti-SRP antibodies. Patient 1 also had anti-TIF1-γ and anti-Ro52 antibodies. The patient 2 had anti-Jo1 and anti-Ro52. The patient 3 had anti-EJ and anti-Ro52 additionally (Fig. 1).

**Fig. 2:**
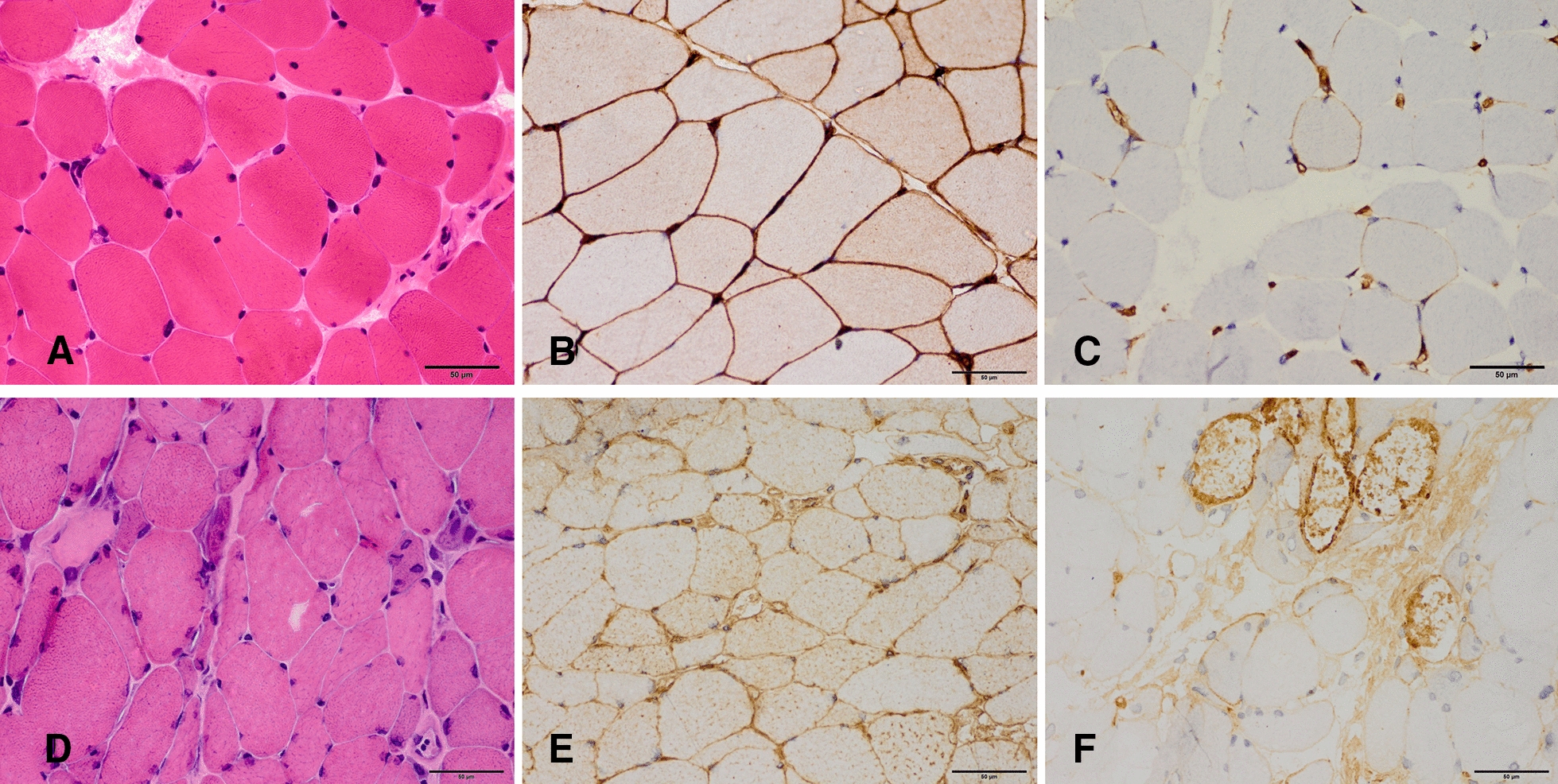
Muscle biopsy from patient 1 and patient 3. Patient 1: **A** hematoxylin and eosin (HE) staining showing no necrotic or regenerating myofibers. **B** Major histocompatibility complex 1 (MHC-I) immunostaining (brown) is upregulated in both the sarcolemma and sarcoplasm of myofibers. **C** Membrane attack complex (MAC) immunostaining showed deposition of MAC on scattered myofibers. Patient 3: **D** HE staining showing many necrotic and regenerative muscle fibers without endomysia inflammatory infiltrates. **E** MHC-I is upregulated in both the sarcolemma and sarcoplasm. **F** Deposition of MAC on non-necrotic myofibers (bars 50 μm, **A**–**F**)

Muscle biopsy revealed general up-regulation of myofiber MHC-I and deposition of MAC on scattered non-necrotic myofibers in patient 1 without obvious necrotic or regenerating myofibers; many necrotic and regenerative muscle fibers without endomysia inflammatory infiltrates was showed in patient 3, with MHC-I upregulated in both the sarcolemma and sarcoplasm and deposition of MAC on non-necrotic myofibers (Fig. 2).

The main treatments in these patients were including: oral prednisolone in all these patients, cyclophosphamide in patient 2, intravenous immune globulin in patient 3. The symptoms of muscle weakness and joint pain were fully recovered in patient 1 after 3 months. The symptom of dyspnea in patient 2 was partially improved and maintain stable during the 3-year follow-up. The muscle weakness deteriorated in patient 3 and eventually died of respiratory failure in one month.

## Discussion

In this study, we report three patients with anti-SRP antibodies and another MSA including anti-TIF1-γ, anti-Jo1 and anti-EJ antibodies, respectively. To our knowledge, this is the first report of anti-SRP and anti-TIF1-γ or anti-EJ co-positive in IIM. In the few reported cases who had multiple MSAs, they all had anti-SRP antibodies, and the most common co-occurring antibodies are anti-synthetase antibodies as in our patients (Table 1) [5–10].

Anti-TIF1-γ antibodies are the most commonly identified in patients with juvenile dermatomyositis with classic dermatomyositis rashes and mild muscle disease [15]. Anti-TIF1-γ antibodies are strongly associated with malignancy in adults more than 40 years of age [16]. Patient 1 with anti-SRP antibodies plus anti-TIF1-γ antibodies showed mild muscle involvements with normal CK levels and not rashes, which seems hard to categorize as anti-SRP syndrome or anti-TIF1-γ syndrome. However, up-regulation of myofiber MHC-I and deposition of MAC on scattered non-necrotic myofibers in patient 1 indicating the possibility of anti-SRP syndrome. Considering the presentation of obvious joint pain, anti-SM antibodies, follow-up study is needed to rule out the diagnosis of overlap myositis [17, 18].

The two patients with anti-SRP antibodies and anti-synthetase antibodies (including anti-Jo1 and anti-EJ antibodies) all had interstitial lung disease as in the reported cases [5–9]. In patients with anti-SRP antibodies, the frequencies of fever, skin rash, arthritis, Raynaud phenomenon and interstitial lung disease were generally low. Interstitial lung disease was happened in 13–22% patients, all of whom had generally mild respiratory symptoms and no correlated to poor neurological outcome [2, 3]. However, arthralgia (75%) and interstitial lung disease (69–80%) are the most prevalent clinical signs associated with anti-synthetase antibodies (including anti-Jo1, anti-PL7, anti-PL12, anti-OJ, and anti-EJ) [13, 14]. The frequency of ILD in these patients with anti-SRP and anti-synthetase antibodies is higher than the results reported above. It remains unclear whether co-occurred with anti-SRP antibodies aggravate the development of ILD. However, these patients with ILD may need more aggressive treatment. Therefore, in these patients with anti-SRP antibodies and another anti-synthetase antibodies, the clinical characteristics with more extra-muscular signs including prominent ILD tend to be anti-synthetase syndrome especially in patient 2 due to anti-Jo1 antibody. In histology, perifascicular necrosis, the characteristic finding of anti-synthetase syndrome [14], was not found in patient 3, whose muscle biopsy results tend to be necrotizing myopathy according with anti-SRP syndrome [1, 2]. Thus, the symptoms of patient 3 might match both anti-synthetase and anti-SRP syndrome, suggested that this combination of antibodies probably portends a more severe disease with more high frequency of ILD, which might be associated with a poor prognosis.

There were some limitations in our study. Firstly, this study is a retrospective summary of a small number of clinical cases, and more studies are needed to reveal the clinical phenotypic characteristics when multiple myositis-specific antibodies coexist. Secondly, the antibodies in these cases were detected only using commercial line immunoblot assay and not confirmed by other validated immunoprecipitation methods to rule out assay error. Only patient 2 had a repeat antibody test with similar results, and although considering the possibility of false positives due to technical problems was minimal, other patients were not retested due to no retained blood samples.

In conclusion, our findings provide more evidence that the coexistence of anti-SRP antibodies and another one MSA may lead to various clinical symptoms, which may mainly cause by one of the MSAs, namely the responsible antibody, or interact in a complex syndrome, thus expanding the clinical spectrum of idiopathic inflammatory myopathy.

## Data Availability

The datasets used and analyzed during the current study available from the corresponding author on reasonable request.

## Abbreviations

MSAs: Myositis-specific autoantibodies
SRP: Signal recognition particle
IIM: Idiopathic inflammatory myopathy
MAAs: Myositis-associated antibodies
IMNM: Immune-mediated necrotic myopathy
CK: Creatine kinase
TIF1-γ: Transcriptional intermediary factor 1-γ
CT: Computed tomography
ANA: Antinuclear antibody
dsDNA: Double-stranded DNA
ENA: Extractable nuclear antigen
MHC-I: Major histocompatibility complex 1
MAC: Membrane attack complex
